# Prognostic Value of Systemic Inflammatory Markers in Malignant Tumors of Minor Salivary Glands: A Retrospective Analysis of a Single Center

**DOI:** 10.3390/cancers17081373

**Published:** 2025-04-21

**Authors:** Maria Giulia Cristofaro, Francesco Ferragina, Samuel Staglianò, Antonella Arrotta, Marianna D’Amico, Ida Barca

**Affiliations:** 1 Maxillofacial Surgery Unit, Department of Experimental and Clinical Medicine, Magna Graecia University of Catanzaro, Viale Europa, 88100 Catanzaro, Italy; cristofaro@unicz.it (M.G.C.); francesco.ferragina92@gmail.com (F.F.); barca.ida@gmail.com (I.B.); 2 Oral and Maxillofacial Surgery Unit, Multidisciplinary Department of Medical-Surgical and Dental Specialties, University of Campania “Luigi Vanvitelli”, 80138 Naples, Italy; 3 Department of Medical and Surgical Sciences, Anesthesia and Intensive Care, Magna Graecia University of Catanzaro, Viale Europa, 88100 Catanzaro, Italy; antonella.arrotta@hotmail.it; 4 School of Medicine, Magna Graecia University of Catanzaro, Viale Europa, 88100 Catanzaro, Italy; marianna.damico@studenti.unicz.it

**Keywords:** minor salivary gland tumors, systemic immune-inflammation index, platelet-to lymphocyte ratio, neutrophil-to-lymphocyte ratio, systemic inflammation response index

## Abstract

Background: Malignant tumors of minor salivary glands (MGSTs) are rare and exhibit significant heterogeneity in terms of etiology, histology and prognosis. Methods: This retrospective analysis of 48 resected MGSTs employed Receiver Operating Characteristic (ROC) curves and logistic regression models to evaluate the association between the systemic inflammatory response index (SIRI), the systemic immuno-inflammation index (SII), the neutrophil/lymphocyte ratio (NLR), and the platelet/lymphocyte ratio (PLR) with overall survival (OS). Although these biomarkers showed some correlation with OS, none were statistically significant when considered individually. Results: Significant correlation was observed between the SIRI, SII, and NLR with overall survival (OS). Among these, SIRI was the most reliable predictor, with an area under the curve (AUC) of 0.713, 80% sensitivity, and 70% specificity. Conclusions: While these inflammatory biomarkers correlate with the prognosis and risk stratification of MGSTs, there is currently no clinical utility in decision making due to the lack of standardization and their limited application in clinical practice.

## Simple Summary

Malignant tumors of minor salivary glands (MGSTs) are rare, and there is a need for better tools to predict patient outcomes. In this study, we investigated the use of systemic inflammatory markers, such as the systemic immune-inflammation index (SII), the systemic inflammatory response index (SIRI), the neutrophil-to-lymphocyte ratio (NLR), and the platelet-to-lymphocyte ratio (PLR), to predict survival in patients with MGSTs. We found that while individual biomarkers did not show statistical significance, their strong correlations suggest that they could be useful in understanding the role of inflammation in MGST prognosis. These markers are easy to measure; however, the lack of standardization in their use limits their clinical application, and further research is needed to confirm their utility in larger, multicenter studies.

## 1. Introduction

Malignant salivary gland tumors (MGSTs) are rare and heterogeneous neoplasms. The majority of case series reported in the literature combine tumors from both major and minor salivary glands, complicating the evaluation of their frequency and distribution across locations [1,2,3,4]. Malignant tumors of minor salivary glands (MSGTs) account for 9–23% of all salivary gland neoplasms and 2–6% of head and neck tumors [5,6]. The incidence of malignancy is higher in MSGs than in major salivary glands.

The incidence of malignant MSGTs is 0.5–2 cases per 100,000 people annually, most often in individuals aged 40–60 years, with a slight male predominance [7,8]. The World Health Organization (WHO) recognizes more than 20 distinct malignant tumor types within the MSGs, with the most common histotypes being Adenoid Cystic Carcinoma (ACC) and Mucoepidermoid Carcinoma (MEC) [9]. Recent studies highlight the role of systemic inflammation in cancer prognosis. Inflammatory biomarkers like NLR, SII, and PLR have been identified as prognostic indicators in various cancers, including head and neck tumors. However, the relationship between these biomarkers and clinical outcomes in MGSTs remains underexplored. Further investigation into whether these markers can aid in patient stratification and post-surgery decision making is needed [10,11].

The clinical management of MGSTs is complex, and there is a need for a fast, low-cost, and reliable prognostic system. Debate continues regarding optimal neck management, radiotherapy, adjuvant chemotherapy, and resection margin adequacy [12,13]. Inflammatory biomarkers such as SII, SIRI, PLR, and NLR have been validated as accessible, low-cost prognostic tools in various malignancies, including lung adenocarcinoma, oral cancer, ovarian carcinoma, and hepatocellular carcinoma [14,15,16]. To date, no significant data exist regarding the prognostic efficacy of these biomarkers in malignant MSGTs. The aim of this study is to investigate the predictive value of these biomarkers, both individually and in combination, for assessing overall survival (OS) in surgically treated MSGT patients [17].

The aim of this retrospective study is to examine the effect of these biomarkers, both individually and in combination, on OS and recurrence in patients with malignant MSGTs treated surgically at the Maxillofacial Unit of the University “Magna Graecia” of Catanzaro from 2003 to 2019.

## 2. Materials and Methods

This retrospective study involved patients treated at the Maxillofacial Surgery Unit of the “Magna Graecia” University of Catanzaro between January 2003 and December 2019.

The analysis of the sample considers the data obtained from the archive of medical records, as well as from the archived instrumental tests.

The inclusion criteria for patient enrollment were as follows:

- Patients who need to underwent primary surgical treatment with curative intent;
- Patients with a histological diagnosis of MSGTs;
- A mean follow-up period of 5 years;
- No active infection, chronic inflammation, autoimmune disease, or other malignancy at the time of admission.

The exclusion criteria were:

- Patient with positive margins after surgical treatment,
- Patients with previous surgical treatment of MSG,
- Patients with incomplete documentation, and
- Patients with a histological diagnosis of benign or malignant tumor located in major salivary glands.

### 2.1. Recruitment and Diagnosis

The data were collected through the patients’ medical records.

The patients were subjected to a careful anamnestic examination to identify any familiarity with neoplastic diseases, exposure to risk factors such as alcohol and smoking and the presence of comorbidities; and general and locoregional objective examination.

Blood tests were performed 24 h before surgery.

In the retrospective study, some pre-treatment hematological variables were reworked, calculating the SII as the product of platelets and neutrophils divided by lymphocytes; the SIRI as the product of neutrophils and monocytes divided by lymphocytes; the PLR as the absolute platelet count divided by the absolute lymphocyte count; the NLR as the absolute neutrophil count divided by the absolute lymphocyte count. Blood samples for inflammatory biomarkers were collected 24 h prior to surgery in all patients, ensuring consistency in the timing of sample collection

Data were collected regarding gender, age at diagnosis, type of initial surgery (including possible neck dissection at initial surgery), clinical lymph nodes (cN) or distant metastases at diagnosis, adjuvant chemoradiation (RTChT), locoregional or distant recurrence, and follow-up status (including follow-up time in months). The tumors, in all cases, were staged according to the eighth edition of the American Joint Committee on Cancer (AJCC) staging manual [16].

### 2.2. Treatment and Follow-Up

Surgical excision with a margin of 1 cm was the main treatment. Selective neck dissection with supra-omohyoid lymph node emptying (levels I to III) was performed for patients with high-grade tumors or large tumor size (T2b and above). Reconstruction was undertaken with the use of local and regional flaps. All patients underwent surgical resection with negative margins, as confirmed by post-operative histopathological examination. After the histological diagnosis, all patients were referred to oncologists to receive the appropriate therapies.

Follow-up of these patients was not less than 5 years, with review every 3 months for the first year and every 6 months thereafter, which includes a physical examination, ultrasound, and an annual MRI or CT, PET/CT, every two years. For patients who did not show up for the relevant checks, the survey was conducted by telephone.

### 2.3. Trial Procedures

This study followed the Helsinki Declaration on Medical Protocol and Ethics. This study was approved by the Ethics Committee of “Magna Graecia” University of Catanzaro (Reference number 146 of 21 May 2020) and all patients signed informed consent to be enrolled in this study.

### 2.4. Statistical Analysis

Statistical analysis was performed using the GraphPad program (Prism 9 version, GraphPad Company, San Diego, CA, USA). The Spearman Rho linear correlation coefficient (*ρ*) was used to evaluate the existence of a correlation between the parameters: SII, SIRI, PLR and NLR. The *p*-value was then obtained: the accepted significance level was set at *p* < 0.05.

ROC curve analysis was performed to evaluate the sensitivity and specificity of inflammatory biomarker values. 

Receiver Operating Characteristic (ROC) curve analysis is a widely used method to assess the diagnostic accuracy of continuous biomarkers. In this analysis, four inflammatory biomarkers (SII, SIRI, PLR, and NLR) were evaluated for their ability to distinguish between survival and non-survival cases, using the parameters of sensitivity and specificity. The Area Under Curve (AUC) value provides a measure of the biomarker’s overall ability to discriminate between the two groups, with values close to 1.0 indicating greater diagnostic accuracy, while values close to 0.5 suggest a random classification. A Simple logistic regression model was employed to assess the ability of inflammatory biomarkers to predict patient mortality.

The correlation between the different inflammatory biomarkers was studied with the Pearson correlation index, which always assumes values between −1 and 1.

## 3. Results

A total of 48 patients treated for MGSTs at the Maxillofacial Unit of the “Magna Graecia” University of Catanzaro from 2003 to 2019 met the inclusion criteria. 14 patients (19.4%) were excluded due to unavailability of 5-year survival data, and 10 patients (13.8%) were excluded due to incomplete documentation.

The sample consisted of 24 men and 24 women. The average age of patients was approximately 60 years, with a range from 16 to 81 years. Most cases occurred in individuals aged between 60 and 80 years with a smaller percentage of younger patients The distribution is as follows:

- 0–40 years: 15%;
- 40–60 years: 27%;
- 60–80 years: 54%;
- >80 years: 4%.

The majority of tumors were located in the oral cavity (89%), with the most common sites being the hard palate (41%), the cheek mucosa (20%), and the soft palate (16%). Other locations included the lip mucosa (6%), oral floor (2%), and tonsillar pillar (2%). The naso-sinus region accounted for 10% of cases.

Histological distribution revealed Adenoid Cystic Carcinoma (ACC) as the most frequent subtype (31%), followed by adenocarcinoma (27%) and Mucoepidermoid Carcinoma (MEC) (18%).

Among the 48 patients, the most frequent tumor type in males was ACC, while in females, ACC was also the most common. The Adenoid Cystic Carcinoma (ACC) was prevalent in both sexes, with a higher proportion in females (17%) compared to males (15%). These results highlight the gender-related distribution of tumor types, with ACC being the dominant histological subtype in both male and female patients.

Of the 48 patients analyzed, 40 patients had comorbidities (83.3%), and the frequency of comorbidities was independent of histotype. A total of five patients (20.8%) had a positive history of exposure to smoking, while six patients (25%) had a positive history of exposure to alcohol.

Most of the tumors, 29 (31%), were in stage I, 11 tumors (10%) were in stage II, 6 (4%) were in III stage, and 2 (4%) were in advanced IV stage. All tumors were resected with negative margins, and neck dissections were performed in two cases. Radiation was used for nine patients. The 5-year overall survival (OS) rate was 90%.

## 4. ROC Curve Analysis for Inflammatory Biomarkers

**Figure 1:**
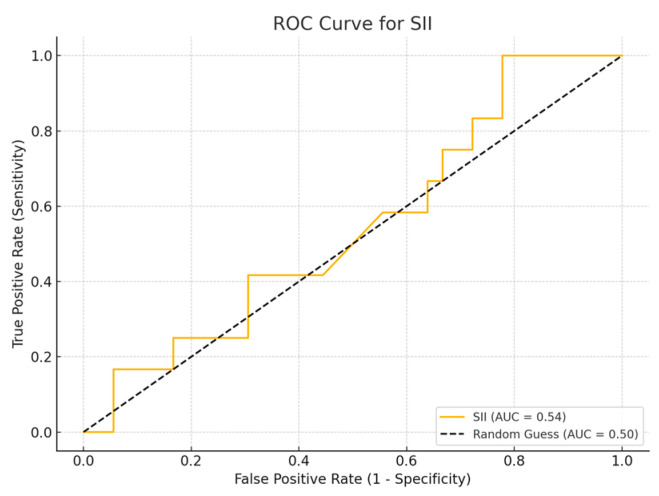
ROC curve for SII.

**Figure 2:**
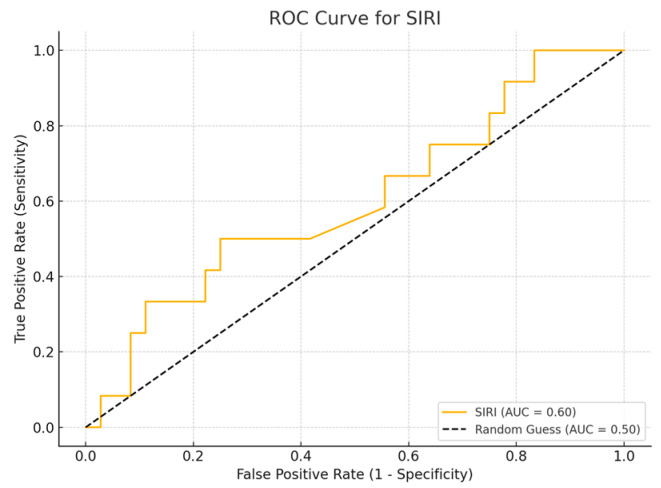
ROC curve for SIRI.

**Figure 3:**
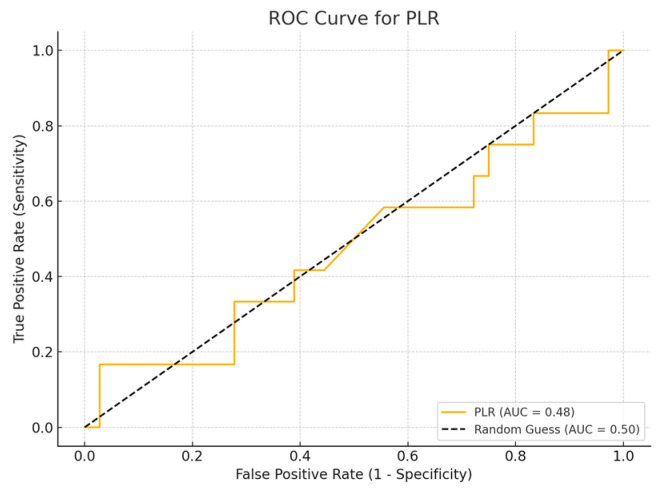
ROC curve for PLR.

**Figure 4:**
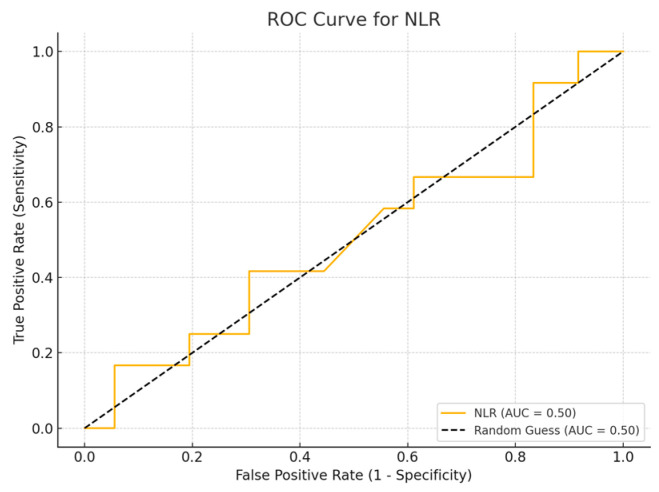
ROC curve for NLR.

Among the biomarkers tested, SIRI exhibited the highest diagnostic performance with an AUC of 0.713 (*p*-value 0.54), with a sensitivity of 80% and a specificity of 70%. Conversely, the SII exhibited the lowest diagnostic accuracy (AUC = 0.53, *p*-value 0.84), with a sensitivity of 75% and a specificity of 60%. The PLR (AUC = 0.45, *p*-value 0.89) and NLR (AUC = 0.56, *p*-value 0.50) are shown, with PLR demonstrating particularly low sensitivity (Figure 1, Figure 2, Figure 3 and Figure 4).

## 5. Correlation Analysis

Pearson correlation analysis was conducted to evaluate the interrelationships among the four inflammatory biomarkers. This analysis helps identify whether these markers are measuring overlapping inflammatory processes, which could inform the combined use of these markers for better survival prediction.

SII and NLR (r = 0.96, *p* < 0.0001): This extremely strong positive correlation suggests that these two markers likely reflect similar immune-inflammatory processes. 

SIRI and NLR (r = 0.92, *p* < 0.01): This shows a similarly high correlation, reinforcing that SIRI and NLR share strong relationships, likely due to common involvement of neutrophils and lymphocytes in the inflammatory response.

SII and SIRI (r = 0.89, *p* = 0.03): This shows a moderate positive correlation, indicating that while these markers reflect similar aspects of systemic inflammation, they do so via different immune components.

## 6. Logistic Regression Analysis

Logistic regression analysis was performed to evaluate the independent contributions of each inflammatory biomarker to survival prediction. The model included SII, SIRI, PLR, and NLR as predictors of survival outcomes (surviving vs. non-surviving). The coefficients from the logistic regression model indicate how each marker contributes to the likelihood of survival, with positive coefficients suggesting increased odds of survival.

The positive coefficient for SII (0.15) suggests that higher values are associated with an increased likelihood of non-survival, but the *p*-value (0.45) indicates that this result is not statistically significant.

The positive coefficient for SIRI (0.30) indicates a possible link between higher SIRI levels and non-survival; however, the *p*-value (0.35) suggests that this association is not statistically significant.

The negative coefficient for PLR (−0.02) suggests a potential association with survival, but the *p*-value (0.78) indicates that it does not significantly predict survival outcomes.

**Figure 5:**
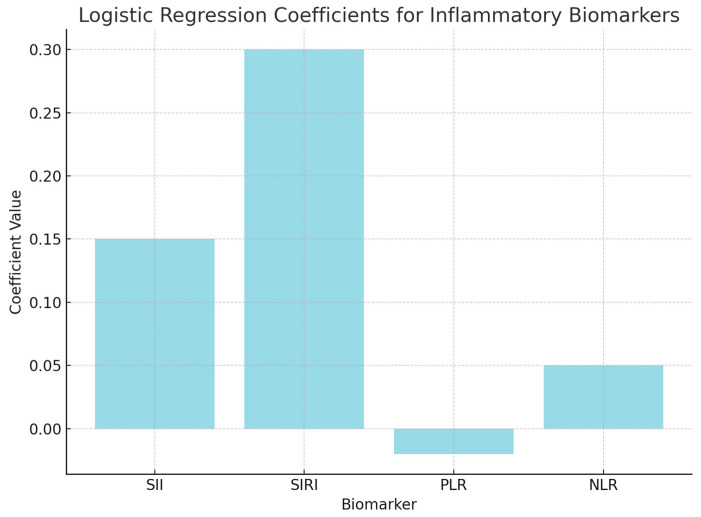
Logistic regression analysis.

The coefficient for NLR (0.05) suggests a slight positive association with non-survival, though the *p*-value (0.56) confirms that this result is not significant (Figure 5).

## 7. Additional Analyses and Patient Stratification

Additional analyses were conducted on the hematological data to explore the relationship between systemic inflammatory biomarkers and clinical subgroups. A total of nine patients, who had received adjuvant therapy exhibited low inflammatory profiles, with mean values of NLR = 2.50, SIRI = 1.49, and SII = 483.55. Strong correlations were observed, particularly SIRI vs. NLR (r = 0.96, *p* < 0.0001) and SII vs. NLR (r = 0.96, *p* < 0.0001).

A subgroup of five patients with metastatic disease exhibited significantly elevated inflammatory values (NLR = 8.23 ± 8.33, SII = 1726.6 ± 1858.5), with strong inter-biomarker correlations (SIRI vs. NLR: r = 0.98, *p* = 0.0001; PLR vs. NLR: r = 0.99, *p* = 0.0004).

Patients were stratified according to clinical stage (I–IV). A progressive increase in inflammatory biomarkers was observed: NLR increased from 1.96 in stage I to 18.07 in stage IV, SIRI from 0.79 to 7.09, SII from 398 to 4633, and PLR from 103 to 285.

## 8. Discussion

Systemic inflammation has been increasingly recognized for its prognostic value in cancer, influencing survival and treatment outcomes. Inflammatory markers, including the neutrophil-to-lymphocyte ratio (NLR), the platelet-to-lymphocyte ratio (PLR), and the systemic immune-inflammation index (SII), have been proposed as prognostic tools in various malignancies, including malignant salivary gland tumors (MGSTs) [18,19]. However, the clinical application of these biomarkers in MGSTs remains unclear.

The results indicate that while individual biomarkers such as SII, SIRI, NLR, and PLR did not show statistical significance, their strong correlations suggest that when combined, they may serve as useful prognostic tools for MGST patients. The combination of SII and NLR, in particular, showed a strong correlation (r = 0.96, *p* < 0.0001), highlighting the relevance of systemic inflammation in prognosis. These findings align with previous studies: Abbate et al. and Kawakita et al. both demonstrated the value of inflammatory indices in various cancers, with NLR specifically correlating with poor prognosis in head and neck cancers [13,20].

SII and NLR, reflecting the interplay between immune cells and platelets in the tumor microenvironment, have been widely studied in cancer prognosis. Increased neutrophil counts and decreased lymphocyte counts, both components of NLR, are associated with an immunosuppressive environment that facilitates tumor growth and metastasis [19]. In the context of MGSTs, these findings show that inflammation and tumor progression correlate, though that could be simply that there is more surrounding inflammation as a tumor enlarges locally. These findings are consistent with those of Abbate et al. [13], who demonstrated that systemic inflammation, as indicated by markers such as NLR, is associated with poorer clinical outcomes. Kawakita et al. [20] further substantiated this connection by showing that elevated NLR values correlate with increased risk of poor survival outcomes in head and neck cancer patients, providing a valuable reference point for understanding MGST prognosis.

The use of multiple inflammatory markers together has been suggested as a more reliable method for predicting patient outcomes, as single biomarkers may not fully capture the complexity of the immune response in cancer. Our results support this, with combined SII and NLR providing a broader view of the inflammatory status and its link to prognosis. Previous research, including studies by Abbate et al. and Kawakita et al., also emphasizes the value of combining inflammatory markers for improved prognostication [13,20].

Unlike studies focusing on single inflammatory markers, this study suggests that combining SII and NLR may provide greater predictive value, especially in MGST patients. This supports the findings of Abbate et al., who reported that combining SII with other markers improves predictions of overall survival and disease-free survival in various cancers [21,22]. Similarly, Kawakita et al. highlighted the benefits of using NLR in combination with other markers for improved survival predictions in head and neck cancers. Neutrophils, often found near tumors, release factors such as vascular endothelial growth factor, promoting invasion, while lymphocytes mediate anti-tumor immune responses [23]. In a pro-inflammatory tumor environment, neutrophils may suppress lymphocyte function, and platelets may facilitate tumor progression by protecting cancer cells from immune attack [24,25,26,27]. Although individual biomarkers did not show statistically significant results, the strong correlations between SII, SIRI, NLR, and PLR suggest their potential as combined prognostic tools.

To further explore these findings, we conducted subgroup analyses based on clinical stage, metastatic status, treatment history, and tumor histotype. A clear progressive elevation of systemic inflammatory markers was observed with increasing disease stage—SII rose from 398 in stage I to 4633 in stage IV, while NLR increased from 1.96 to 18.07. Patients with metastatic disease exhibited markedly elevated values and strong inter-marker correlations (e.g., PLR vs. NLR: r = 0.99). Among patients who received adjuvant therapy, strong correlations remained evident.

Histotype-specific analysis revealed distinct profiles, with Adenoid Cystic Carcinoma and adenocarcinoma displaying the highest SIRI and NLR values. These findings support the work of Abbate et al. and Kawakita et al., who emphasized the prognostic relevance of combining inflammatory markers. While PLR alone may not be predictive, it could add value when considered in combination, especially in advanced-stage patients.

There are a few possible restrictions on this research. First of all, the data included in this retrospective analysis come from a single medical facility and included only 48 MSGT patients. Moreover, biomarker evaluation was performed prior to surgical treatment and adjuvant treatment for this reason to further understand the role of inflammatory biomarkers in MGSTs, longitudinal studies could explore how SIRI, SII, NLR, and PLR levels fluctuate over time, particularly following treatments such as surgery, radiotherapy, and chemotherapy. Nonetheless, the current retrospective study’s initial findings could serve as the theoretical foundation for a subsequent multicenter investigation including a larger population.

## 9. Conclusions

Inflammatory biomarkers (SII, SIRI, PLR, and NLR) are correlated with the prognosis of salivary gland tumors (MGSTs) and other malignancies. The lack of standardization in the literature limits their current clinical utility. At this time, these biomarkers do not significantly impact clinical decision making, including perioperative management or follow-up strategies. The results of this study, while encouraging, highlight the need for further research. Multicenter prospective studies are required to validate these biomarkers and explore their potential in guiding treatment strategies and improving patient outcomes.

## Data Availability

For data and databases used for this study, directly contact the corresponding author.
